# QSPR designer – a program to design and evaluate QSPR models. Case study on pK_a_ prediction

**DOI:** 10.1186/1758-2946-3-S1-P16

**Published:** 2011-04-19

**Authors:** O Skřehota, RS Vařeková, S Geidl, M Kudera, D Sehnal, C-M Ionescu, J Koča

**Affiliations:** 1 National Centre for Biomolecular Research, Masaryk University, Brno, Czech Republic, 625 00, Czech Republic; 2 ANF DATA, a Siemens company, Prague, Czech Republic, 140 00, Czech Republic

## 

Nowadays, a large amount of experimental and predicted data about the 3D structure of organic molecules and biomolecules is available. Advanced computational methods and high performance computers allow us to obtain large sets of descriptors that can be used to estimate physicochemical properties. It is often of interest to study the correlations between descriptors and properties using multilinear regression and to design, parameterize, and test different QSPR (Quantitative Structure Property Relationship) models.

We developed a modular and easily extensible program, called QSPR Designer, which can read or calculate structural properties of atoms and bonds, employ them as QSPR descriptors, and evaluate correlations between the descriptors and the examined physicochemical property of a molecule. Furthermore, the software allows us to effectively design and parameterize QSPR models, calculate physicochemical properties via the models, test the quality of the models, and provide graphs and tables summarizing the results.

The performance of the software is demonstrated by a case study on the prediction of pK_a_. The pK_a_ is of fundamental relevance for chemical, biological and pharmaceutical research, because many important physicochemical properties are pK_a_ dependent. Unfortunately, pK_a_ is also one of the most challenging properties to calculate [1]. Atomic charges have proven very successful descriptors for the prediction of pK_a_[2]. Charges can be calculated using a variety of methods (HF, MP2, functionals, etc.), population analyses (Mulliken, ESP, NPA, etc.) and basis sets. Consequently, the procedure of charge calculation strongly influences their correlation with pKa [3]. Using the QSPR Designer, we have successfully designed, evaluated, and compared 75 different QSPR models for the prediction of pK_a_ from charges. Our best model predicted the pK_a_ for 143 phenols with a correlation coefficient 0.969, RMSE (root mean square error) 0.416 and the average pK_a_ error 0.329.
